# Natural Product Inhibition and Enzyme Kinetics Related to Phylogenetic Characterization for Bacterial Peptidyl-tRNA Hydrolase 1

**DOI:** 10.3390/molecules26082281

**Published:** 2021-04-15

**Authors:** D. Scott Strange, Steven S. Gaffin, W. Blake Holloway, Meredyth D. Kinsella, Jacob N. Wisotsky, Hana McFeeters, Robert L. McFeeters

**Affiliations:** 1 Department of Chemistry, University of Alabama in Huntsville, Huntsville, AL 35899, USA; djs0018@uah.edu (D.S.S.); wbholloway2@gmail.com (W.B.H.); hk0003@uah.edu (H.M.); 2 Department of Biology, University of Alabama in Huntsville, Huntsville, AL 35899, USA; steven.s.gaffin@gmail.com (S.S.G.); mdk0012@uah.edu (M.D.K.); jacobwisotsky@gmail.com (J.N.W.)

**Keywords:** peptidyl-tRNA hydrolase, novel antibiotic target, protein biosynthesis, phylogenetic analysis, natural product inhibition, broad- and narrow-spectrum inhibition

## Abstract

With the relentless development of drug resistance and re-emergence of many pathogenic bacteria, the need for new antibiotics and new antibiotic targets is urgent and growing. Bacterial peptidyl-tRNA hydrolase, Pth1, is emerging as a promising new target for antibiotic development. From the conserved core and high degree of structural similarity, broad-spectrum inhibition is postulated. However, Pth1 small-molecule inhibition is still in the earliest stages. Focusing on pathogenic bacteria, herein we report the phylogenetic classification of Pth1 and natural product inhibition spanning phylogenetic space. While broad-spectrum inhibition is found, narrow-spectrum and even potentially clade-specific inhibition is more frequently observed. Additionally reported are enzyme kinetics and general in vitro Pth1 solubility that follow phylogenetic boundaries along with identification of key residues in the gate loop region that appear to govern both. The studies presented here demonstrate the sizeable potential for small-molecule inhibition of Pth1, improve understanding of Pth enzymes, and advance Pth1 as a much-needed novel antibiotic target.

## 1. Introduction

With the relentless development of drug resistance and re-emergence of many pathogenic bacteria, new antibiotics and new antibiotic targets are in critical need. Bacterial peptidyl-tRNA hydrolase (Pth1) is emerging as a promising new avenue for antibiotic development. Pth1 performs the necessary function of recycling peptidyl-tRNA naturally generated from protein biosynthesis [1,2,3] or the expression of minigenes and short ORFs [4,5,6]. Accumulation of peptidyl-tRNA is toxic to cells, obstructing protein translation and rapidly leading to cell death from tRNA starvation [7]. Whereas Pth1 is essential in a vast majority of pathogenic bacteria [8,9], eukaryotes have redundant peptidyl-tRNA hydrolases that are structurally and mechanistically unrelated, yet complement Pth1 function [9,10]. Thus, inhibiting the essential function of Pth1 in bacteria provides a new avenue for antibiotic development. Interrupting protein translation, but not affecting the ribosome, Pth1 inhibition has many advantages in regards to antibiotic development. Inhibition of protein biosynthesis is a proven antibiotic strategy employed by currently used therapeutics, such as aminoglycosides, tetracyclines, and macrolides which target the ribosome. However, being a new target, Pth1 inhibitors will be effective against drug-resistant bacterial strains. With the redundancy of Pth enzymes in eukaryotes and knock-out of Pth1 in yeast having no effect on viability [10], it is expected that Pth1 inhibitors would demonstrate bactericidal activity without significant eukaryotic cytotoxicity. Similarly, with an estimated order of magnitude fewer Pth1 enzymes compared to ribosomes in a typical bacterial cell [4], there is a considerable stoichiometric advantage to targeting Pth1 over the ribosome. Overall, Pth1 holds great promise as a much needed avenue for new antibiotic development.

**Figure 1:**
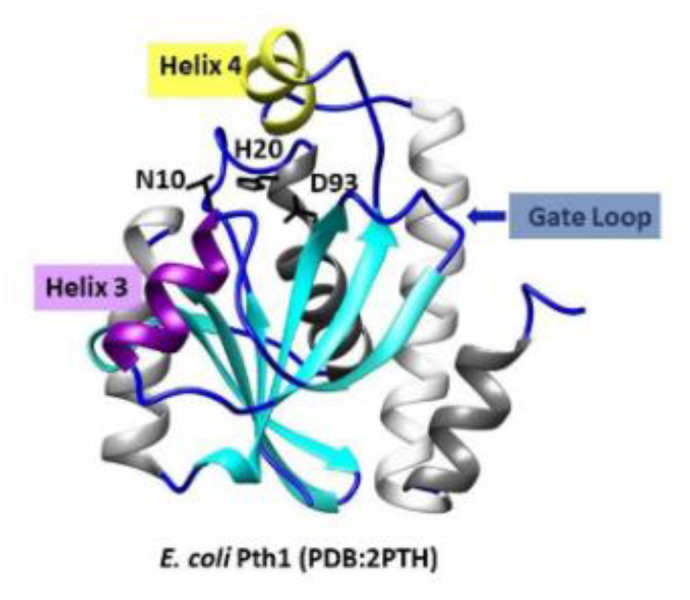
The crystal structure of *E. coli* Pth1 (PDBID: 2PTH). Labeled are the catalytic residues N10, H20, and D93, helix-3 in purple, helix-4 in yellow, and the gate loop. On the right is the amino acid sequence alignment.

An additional advantage to antibiotic development targeting Pth1 is the existing large structural knowledgebase. From the multitude of high-resolution structures reported to date, all have related backbone folds that maintain the recognizable core of α/β-hydrolases. Three key catalytic residues including Asn10, Asp93, and essential His20 (numbering from *E. coli* Pth1, PDBID 2PTH) are found in the active site cleft, see Figure 1 [11]. The active site cleft in Pth1 enzymes is formed by a base loop, a gate loop, and a lid loop. The base loop forms one side of the cleft while the lid and gate loop form the opposite side of the cleft. Dynamic mobility in the microsecond–millisecond range has been reported for the region surrounding the active site in Pth1 from *Mycobacterium tuberculosis* and *Vibrio cholerae* [12,13]. The helix-4 loop serving as a lid over the active site was shown to be the most dynamic, while the gate loop region and helix-3 were more rigid. The binding of partial or mini-substrates along with computational docking provided preliminary insight into substrate recognition [14,15,16,17,18]. A crystal structure of *E. coli* Pth1 in complex with the non-peptide attached TΨC loop of tRNA^Ala^ [16] and small-angle neutron scattering characterization of a catalytically inactive *E. coli* Pth1 mutant bound to heterogeneous peptidyl-tRNAs [17] provided some understanding of selectivity and specificity for peptidyl-tRNAs. Furthermore, past results using tRNA mimics missing the TΨC arm resulted in *E. coli* Pth1 having a 10-fold loss in catalytic efficiency, but the enzyme retained the ability to hydrolyze peptidyl-tRNA [14]. From early enzyme kinetic studies, the length of the substrate peptide moiety, increasing up to four amino acids, correlates with higher affinity K_m_ values [19]. While structure/function studies continue to improve understanding of Pth1 enzymes, no high-resolution structure of a Pth1:peptidyl-tRNA complex has been reported to date.

Complementing structural studies but still in its infancy, small-molecule inhibition of Pth1 is emerging [20,21]. Particularly enabling are advances in functional assays [18,22] and fluorescent substrates for potential high throughput screening [23]. Even with modest throughput methods, a diversity of natural products and synthetic compounds have been shown to inhibit Pth1 [17,24,25]. Thus it appears there are enormous reservoirs of small molecules that may serve as rich sources of lead compounds against this novel antibiotic target. Since a significant proportion of new drugs against infectious disease originate from natural sources [26,27], tremendous potential exists for small-molecule Pth1 inhibition.

Herein we report the phylogenetic classification of Pth1 from pathogenic bacteria and quantitate inhibition of a common panel of natural product extracts against Pth1s spanning phylogenetic space. We also report enzyme kinetics and in vitro protein properties that correlate to phylogenetic clades. Phylogenetic characterization shows that Pth1 enzymes occupy three distinct phylogenetic clades which are related to Gram specificity. Natural product inhibition demonstrates the possibility for broad as well as narrow-spectrum, and, potentially, clade-specific inhibition. Kinetic analysis further highlights the differences between phylogenetic clades, with common V_max_ but differing K_m_ values. Utilizing site-directed mutagenesis, residue differences in the gate loop were shown to affect kinetics and general properties of Pth1s from different clades. This is the first large-scale phylogenetic classification of bacterial Pth1 and the first report of observed narrow-spectrum Pth1 inhibition. This study demonstrates how including homologs spanning phylogenetic space adds important dimensions to antibiotic development and that quite different small-molecule inhibition can be observed for highly conserved enzymes. Overall, the results presented here are important steps for continued antibiotic development targeting Pth1.

## 2. Results

### 2.1. Phylogenetic Tree

**Figure 2:**
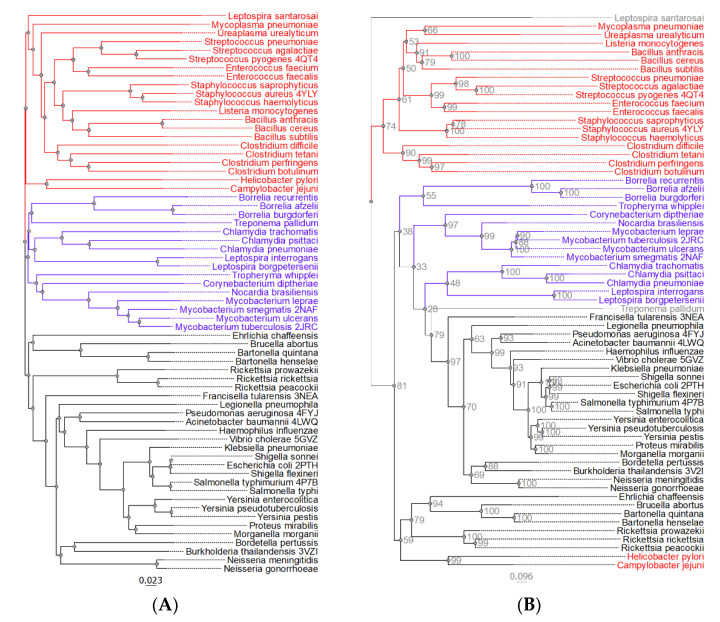
The Phylogenetic Relationships of Pth1 from Pathogenic Bacteria. (**A**) Shown are the simple phylogenetic relations for Pth1 from pathogenic bacteria. The intermediary Clade 2 is colored blue. Pth1 with known structures have the 4 character PDBID after the name. (**B**) Shown is the phylogenetic tree generated in IQTREE [28,29] with bootstrap values indicated. Coloring reflects grouping identified in the simple phylogenetic analysis. Bootstrap values are shown in gray.

While the phylogenetic analysis focused on pathogenic bacteria, Pth1 from three relevant non-pathogenic bacterial species were also included. Specifically, Pth1 from *Bacillus cereus*, *Bacillus subtilis*, and *Mycobacterium smegmatis* were included since they are commonly used as substitute models for related, highly pathogenic species. Simple phylogenetic characterization of Pth1 revealed three major clades, see Figure 2A. Clade composition correlated with Gram specificity. While Clade 1 is strictly populated by Pth1 from Gram-negative bacteria (like *E. coli* Pth1), Pth1 from Gram-positive bacterial species populate Clade 3. Clade 2 is intermediate, composed of Pth1 from both Gram-positive and Gram-negative bacteria along with several Gram-indistinct members. The theme of Clade 1 being distinct from Clade 3, with Clade 2 being intermediary, holds true for several other Pth1 properties, presented below. 

The initial phylogenetic tree was subject to a bootstrap analysis that revealed that the clade separation may not be very robust. Therefore, a more sophisticated phylogenetic approach [28,29] was used that produced a tree, in which a majority of the guide tree associations are retained and the bootstrap values better supported the branch separation. However, unlike in the initial phylogenetic tree, only two main clades exist, Figure 2B. The new Clade 1 contains predominantly Pth1 from Gram-positive species and its composition is the same as in the initial guide tree with *H. pylori* and *C. jejuni* Pth1 no longer part of the clade. Since these two species are Gram-negative, it appears that the inclusion of their Pth1 enzymes in the large second clade fits with the Gram-positive/Gram-negative separation. The larger clade, Clade 2, is composed of Pth1 from primarily Gram-negative bacterial species. The division between Clade 2 and 3 from the initial guide tree is not as pronounced, however clear separation between the two groups is still retained. The observation that members of Clade 1 are distinct from Clade 3 (using the simple phylogenetics notation) holds, with members from Clade 2 still being intermediate.

In regards to the solubility of Pth1, it was generally found that the Clade 1 homologs expressed well and were highly soluble under typical conditions. Clade 3 homologs were not very soluble and expression conditions needed to be optimized for the production of significant amounts of soluble recombinant protein. Interestingly, the Clade 2 homolog *M. tuberculosis* Pth1, demonstrated intermediate solubility, between those generally found for Clade 1 and Clade 3 homologs. 

**Figure 3:**
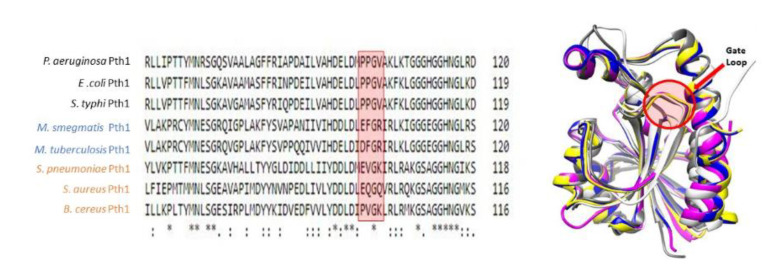
Gate Loop Region of Bacterial Pth1. (**Left**) Multiple sequence alignment of the gate loop region reveals a conserved motif within phylogenetic clades. * indicates identicle residues. (**Right**) The gate loop structure maintains a highly conserved fold following superimposition of crystal structures.

**Figure 4:**
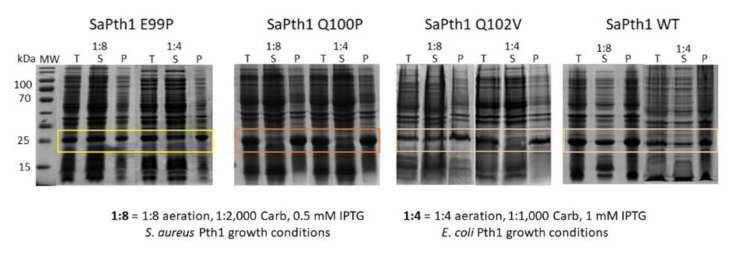
Gate Loop Residues Affect Pth1 Solubility. Solubility for residue changes in the Gate Loop are shown. Aeration is defined as the ratio of volume of culture media to flask volume. The total protein (T), soluble fraction (S) and insoluble pellet (P) are shown for each construct and condition.

Upon inspection of the aligned Pth1 amino acid sequences, one of the most notable differences between the clades was in the gate loop region, see Figure 3. In order to determine the effect of these amino acid differences in the gate loop, site-directed mutagenesis was utilized. *S. aureus* Pth1 (Gram-positive, Clade 3) gate loop region residues were individually mutated to those of *P. aeruginosa* Pth1 (Gram-negative, Clade 1). The following three mutants were produced: E99P, Q100P, and Q102V. Glycine 101 is conserved, so was not altered. Of note, G101D in *E. coli* Pth1 imparts temperature sensitivity [30]. Unlike wild-type *S. aureus* Pth1 that is expressed relatively insoluble under typical conditions, *P. aeruginosa* Pth1 is quite soluble. Thus we characterized solubility for all three gate loop mutations. E99P showed a trend towards increased solubility (*p*-value of 0.06) when grown and induced for both typical and *S. aureus* optimized growth conditions, see Figure 4. This is evident from the increased intensity of Pth1 in the soluble fraction compared to the pellet. The Q100P mutation resulted in little to no soluble protein. It is speculated that disruption of the Q100-D154 salt bridge unique to *S. aureus* Pth1 is the cause. Q102V did not appear to have any effect on solubility and only the atypical wild-type *S. aureus* Pth1 growth conditions produced soluble protein.

### 2.2. Pth1 Natural Product Inhibition

**Figure 5:**
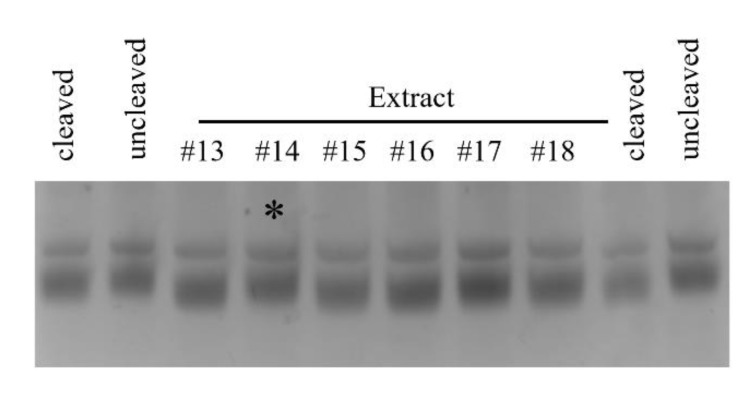
Natural Product Inhibition of *B. cereus* Pth1. Shown is an example of typical inhibition data, cleaved represents the positive control containing fully active Pth1 with no inhibitor. Uncleaved is the negative control, substrate peptidyl-tRNA with no Pth1. The * indicates inhibition by the natural product extract. The lower band is the substrate peptidyl-tRNA that exhibit migration differences based on cleavage state. The top band is 5S rRNA, an artifact of purification, that serves as an internal standard.

**Figure 6:**
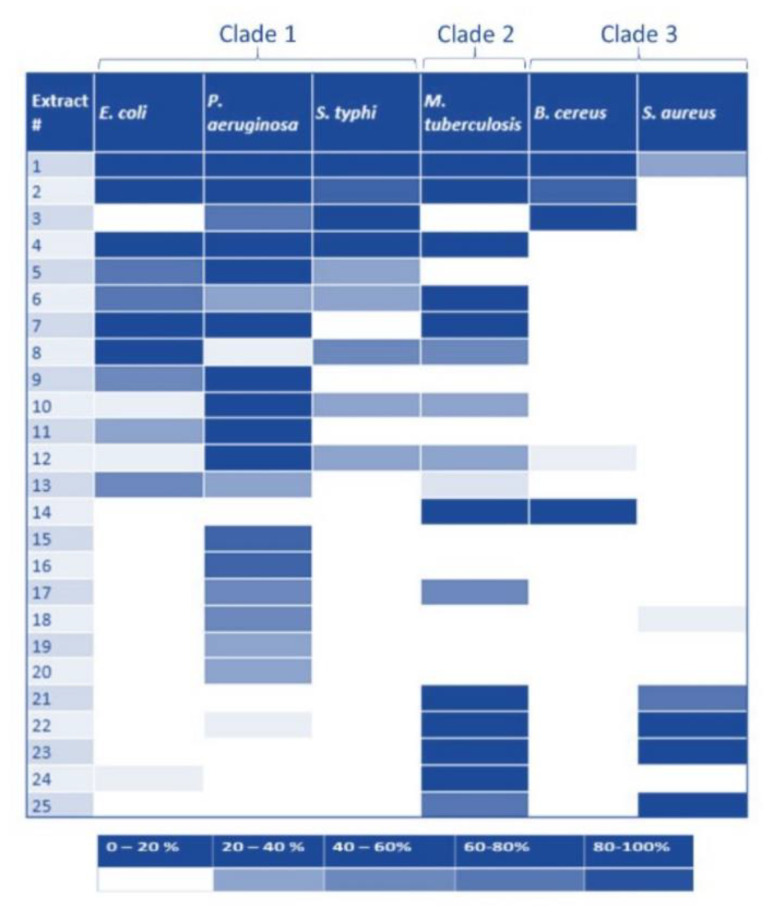
Natural Product Inhibition of Pth1 Spanning Phylogenetic Space. Inhibitory activity for a panel of natural product extracts tested for inhibition against Pth1 is shown. The degree of inhibition is shown at the bottom, with darker color indicating stronger inhibition.

Given the past success of inhibiting Pth1 [18,24,25] along with progress made to facilitate the discovery of the active compounds [31], we utilized natural products to qualitatively and semi-quantitatively characterize Pth1 inhibition, see Figure 5. Using a common panel of natural product extracts, inhibition of substrate cleavage was determined for Pth1 from the following species: *Escherichia coli*, *Pseudomonas aeruginosa*, *Salmonella typhimurium* (Clade 1), *Mycobacterium tuberculosis* (Clade 2), and *Bacillus cereus* and *Staphylococcus aureus* (Clade 3), see Figure 6. Of immediate interest, very different inhibition profiles were found for Pth1 from different pathogenic bacteria. While a finite number of homologs were tested for each clade and acknowledged for the following interpretation, the differences between homologs are clear. For some natural product extracts, like *Ardisia compressa* (extract #3), *Mandevilla veraguasenis* (#4), *Exothea paniulata* (#5), and *Inga sierra* (#6), Pth1 inhibition strictly followed the clade boundaries. These extracts only inhibited Clade 1 enzymes and the Clade 2 homolog Pth1 from *M. tuberculosis*. Extracts from *Acacia aulacocarpa* (#15), *Ocotea floribunda* (#16), and *Urera caracasma* (#18) demonstrated more narrow-spectrum activity, inhibiting just PaPth1 within Clade 1. Similarly, the *Albizia adenocephala* extract (#14) inhibited Pth1 from *B. cereus* and *M. tuberculosis*. From the susceptibility standpoint, Pth1 from *B. cereus* and *S. aureus*, both Clade 3 members tested, were the least susceptible Pth1 homologs to natural product inhibition, being inhibited by four and six natural products respectively. The most susceptible was Pth1 from *P. aeruginosa*, inhibited by 19 natural products. While it must be kept in mind that the inhibitory activity may not be due to a single component and that a limited number of Pth1 homologs were tested these results provide the first indication that narrow-spectrum Pth1 inhibition is possible and an evidence-based understanding of Pth1 small-molecule inhibition, directly countering the long-held idea that Pth1 sequence homology leads to uniform small-molecule inhibition.

### 2.3. Enzyme Kinetic Parametrization

**Figure 7:**
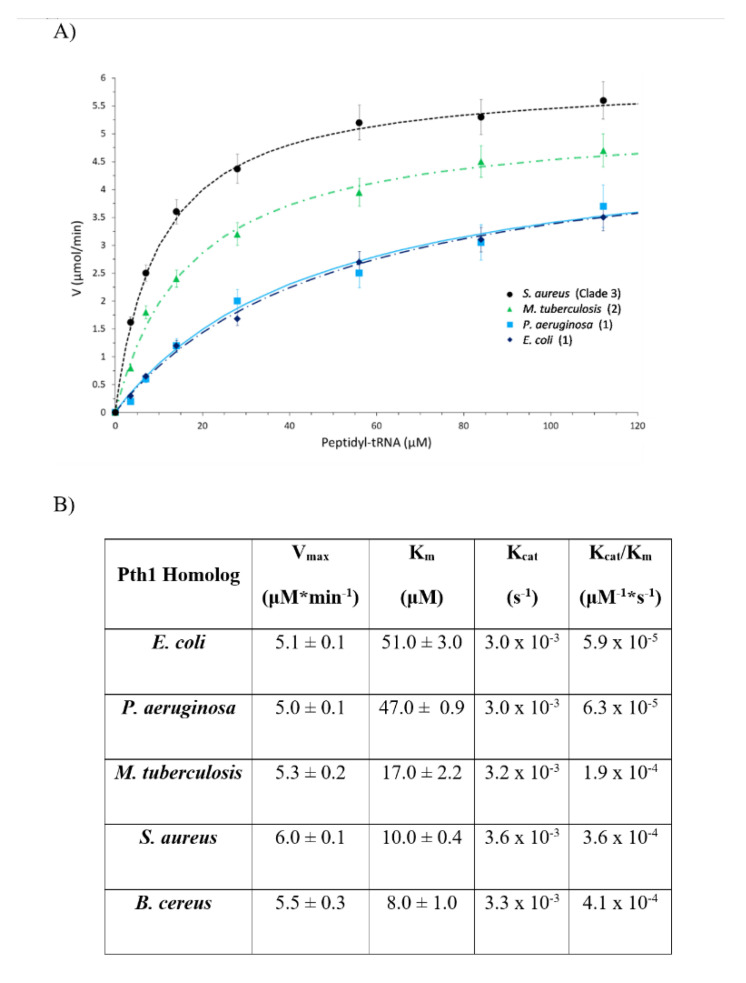
Comparison of Kinetic Parameters for Pth1 from Different Phylogenetic Clades. (**A**) Plots of enzyme velocity versus substrate concentration are shown for Pth1 from *E. coli*, *P. aeruginosa*, *M. tuberculosis*, and *S. aureus*. The representative clade is indicated in parenthesis. (**B**) Table showing a summary of kinetic parameters for Pth1 tested.

**Figure 8:**
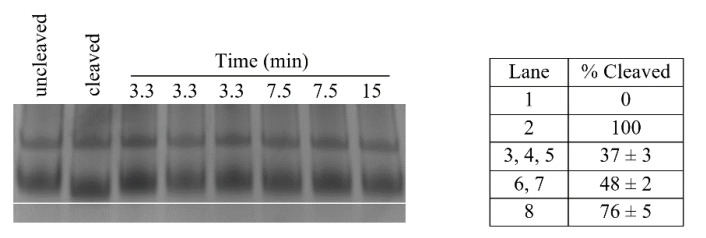
*S. aureus* Pth1 Kinetic Data. Shown is a representative time course of substrate cleavage by Pth1 from *S. aureus*. Uncleaved indicates the negative control, enzyme substrate or peptidyl-tRNA. Cleaved indicates the positive control, enzyme product or peptidyl-tRNA fully cleaved to free tRNA. A white line was added to better visualize the band shift. To the right is a table that shows the percent of substrate cleaved ± standard deviation, calculated from migration distance.

**Figure 9:**
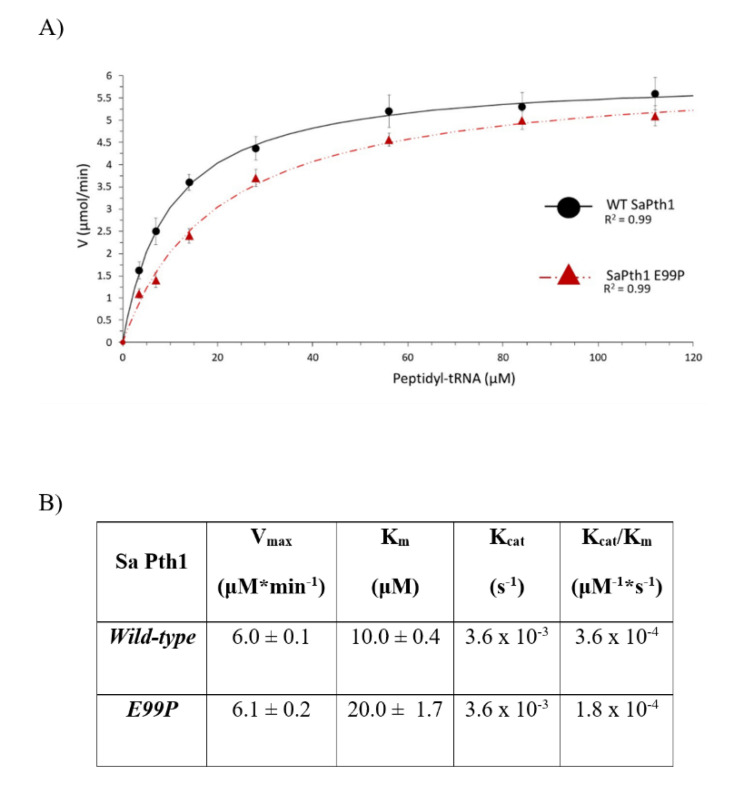
*S. aureus* Pth1 Gate Loop Mutation Affects K_m_ but not V_max_ or K_cat_. (**A**) The velocity of substrate cleavage for wild-type *S. aureus* Pth1 (black circles) is compared to the mutant E99P *S. aureus* Pth1 (red triangles). (**B**) Table showing a summary of kinetic parameters.

The enzyme kinetic parameters also show a correlation to clade boundaries, see Figure 7 with typical data shown in Figure 8. For the Pth1 homologs tested it is clear that Clade 1 representatives have the lowest V_max_, highest K_m_, and lowest K_cat_/K_m_ with the opposite holding true for Clade 3 homologs. As for the phylogenetic characterization, Clade 2 Pth1 kinetic parameters are intermediate between Clade 1 and Clade 3 homologs. While it appears the K_cat_ is similar for all Pth1s, K_m_ is an order of magnitude different with averages of 49 μM for Clade 1, 17 μM for Clade 2, and 9 μM for Clade 3. The differences in kinetic parameters between Clade 1 Pth1s and Clade 2 Pth1s as well as Clade 1 Pth1s and Clade 3 Pth1s were found to be statistically significant with a *p*-value of 0.02 and 0.05 respectively. The difference between Clade 2 and Clade 3 homologs was characterized by a *p*-value of 0.06. Although only relative comparisons can be made due to the use of partial substrates and different assay conditions, the same trends in kinetic parameters were previously found for *E. coli* Pth1 [14,19], *M. tuberculosis* Pth1 [32], and *S. aureus* Pth1 [23]. Given the distinct amino acid differences in the gate loop region and effects on solubility, the E99P (*S. aureus* to *P. aeruginosa*) change was also examined for effects on kinetic parameters, see Figure 9. K_m_ for this mutant was only half that of wild-type *S. aureus* Pth1 whereas V_max_ was relatively unchanged, resulting in a relatively low *p*-value of 0.09 when comparing K_cat_/K_m_. This demonstrates, at least in part, the effect residues in the gate loop have on enzymatic activity. 

## 3. Discussion

In the past, interest in Pth1 as an antibiotic target was mitigated by the high degree of amino acid sequence homology and the accompanying supposition that only broad-spectrum inhibition was possible. Further, given that there exists a human homolog, it was supposed that cytotoxicity would be a significant issue due to the essential function of Pth1 in bacteria. Subsequently, yeast knockout studies showed recycling peptidyl-tRNA in eukaryotes is not critically dependent on Pth1 [10]. Herein we further understanding Pth1 and, for the first time, demonstrate that Pth1 from dissimilar bacterial species can be differentially inhibited by small molecules. Thus, the possibility for narrow-spectrum inhibition and considerable potential for small-molecule inhibition exists, both advancing Pth1 antibiotic development.

Phylogenetic characterization of Pth1 reveals the presence of at least two distinct clades. A correlation between clade and Gram specificity is present, but it is unclear as to the origin. One possibility is tRNA methylation, which is known to be different between Gram-positive and Gram-negative bacteria [33,34,35,36]. However, it is not clear that the methylation affects the acceptor stem which appears to be most closely involved in substrate binding. Yet, at least one Gram-positive bacterial species is known to possess methylation in the acceptor stem [37]. While this may lend itself to recognition via a scanning mechanism [16] with methylation affecting binding, the validity of this model still needs proof in an in vivo setting. Given that the peptidyl-tRNA used in these studies are from a Gram-negative strain, it would be of interest to characterize kinetics using peptidyl-tRNA from a Gram-positive source. 

While the Gram specificity may be unclear, it is clear that the residues in the gate loop are largely responsible for differences in K_m_. Homologs with acidic aspartate or glutamate residues (Clades 2 and 3) versus proline (Clade 1) at position 99, had relatively smaller K_m_ values equating to a higher affinity interaction and improved catalytic efficiency. This may be a result of dynamic constraints caused by the proline residue or negative charge of the acidic residues, though further studies will be needed to clarify this issue. 

In regards to small-molecule inhibition, all Pth1 homologs tested show some degree of narrow-spectrum inhibition. This counters the assumption that the high degree of sequence homology would lead to similar small-molecule inhibition. Moreover, it underscores the advantage of studying homologs from multiple species to get a better understanding of the enzyme family as a whole, not relying on one sequence and extrapolation. Inhibition following clade boundaries could also be distinguished. Regrettably, homologs in Clade 1 (Gram-negative) were most susceptible to inhibition, whereas Clade 3 homologs (Gram-positive) showed the least susceptibility. While this has nothing to do with the more abundant extracellular peptidoglycan, it correlates with the greater difficulty of targeting Gram-positive bacteria with small-molecule inhibitors. The differences in clade inhibition do open other possibilities. While purely speculative, one could imagine engineering major gut species to have Pth1 from both Clade 1 and Clade 3. Thus when administering Pth1 antibiotics, one could at least partially circumvent the side effects of eliminating beneficial species in the host GI tract by also administering probiotics with the other Clade Pth1 (that would be resistant to the systemic Pth1 antibiotic targeting the pathogen). Regardless, these findings demonstrate the potential not only for broad-spectrum inhibition of Pth1, but also narrow-spectrum inhibition.

The enzyme kinetics characterization was performed using bulk peptidyl-tRNA, the same substrate as that for inhibition studies. Thus, the results are interpreted with the caveat that a mixture of tRNAs with variable peptides was used. Nevertheless, the very similar K_cat_ values for homologs across phylogenetic space indicate the chemistry of the reaction was mostly unchanged. This is expected given the absolutely conserved catalytic histidine and highly conserved supporting residues. However, K_m_ and therefore K_cat_/K_m_ for each homolog do show significant differences between Clade 1 and Clade 3. K_m_ and K_cat_/K_m_ for the *M. tuberculosis* Pth1, the only Clade 2 representative, are intermediate, between Clade 1 and 3 values. These findings and the overall trend in K_m_ are consistent with previously published results [14,23,32].

Overall, it is clear that there are significant differences between Pth1 across bacterial species. Even with the high degree of sequence homology, the different kinetic parameters indicate that while V_max_, and thereby the catalytic reaction, is mostly unchanged, substrate binding is different given the large differences in K_m_. The difference in binding substrate agrees with the differential small-molecule inhibition observed for natural products. These observations give a clear indication that selective Pth1 inhibition is possible, elevating Pth1 as a viable antibiotic target.

While the structural knowledgebase of Pth1 now spans phylogenetic space, the majority of crystal structures come from Clade 1 Pth1 enzymes. Additionally, the resolution of crystal structures from Clade 1 Pth1 (typically below 1.7 Å) is considerably better than for those from Clade 3 (2.7 and 2.2 Å). A possible explanation may be the clade-specific correlation with solubility so simply explained by the ease of preparation (from our personal experience and as intuited from the literature). The difficulty and lower resolution of structures from Clade 3 may also be attributed to the gate loop. If the presence of a proline alters dynamics or impacts sampling of conformational space, differences in crystallization and uniformity of crystallization can be readily explained. This may also be related to the difficulty in crystallizing Pth1 with small-molecule inhibitors bound. We observed that soaking in does not lead to occupancy in the binding site, even for known competitive inhibitors. Similarly, co-crystallization significantly changes the crystallization conditions. Thus it appears a lock-and-key fit is not supported for Pth1. This also agrees with recent preliminary computational efforts for which high throughput screening to the apo structure does not lead to any significant enrichment of higher affinity binding. Nor does the computational docking of known Pth1 inhibitors agree with experimentally derived binding affinities. Thus it is suspected that a yet unknown conformation change occurs upon high-affinity binding in the active site. 

## 4. Materials and Methods

### 4.1. Phylogenetic Characterization

Using the EMBL-EBI bioinformatics tools [38], multiple amino acid sequences were aligned using MUSCLE [39] and simple phylogenetic analysis was performed by the neighbor-joining method [40]. More in-depth phylogeny was obtained using IQ-TREE [28,29] that employed ModelFinder [41] to find the best substitution model. Bootstrap values were determined from 1000 replicates by the ultrafast bootstrap [42]. SH-aLRT analysis [43] was also performed. The Newick format generated trees from both tools were visualized in UGENE [44].

### 4.2. Pth1 Expression and Purification

Pth1 was expressed and purified as previously reported [12,45,46,47]. Briefly, for all constructs other than *M. tuberculosis* that was a kind gift from Dr. Vijayan [48], the *E. coli* codon-optimized Pth1 sequence was cloned into a pKQV4 expression vector which introduced an N-terminal hexahistidine tag. After transformation into chemically competent BL21(DE3)pLysS *E. coli*, individual colonies were selected from plates. Cells were typically grown at 37 °C in LB media containing 100 mg/mL carbenicillin, induced with 1 mM (final concentration) IPTG at an OD_600_ of 0.7, and harvested after 6 h. However, to obtain soluble *S. aureus* Pth1, conditions were optimized to 20 mg/mL carbenicillin, 0.5 mM IPTG at and induction OD_600_ of 0.9, the post-induction temperature was shifted to 12 °C, and cells were harvested after 16 h. Cells were then pelleted and stored frozen at −80 °C until lysis and purification via metal chelation chromatography using a Ni^2+^ bound His-Trap Fast Flow column (GE Lifesciences, Pittsburgh, PA, USA). Purified Pth1 was dialyzed in the appropriate buffer and quantified using UV spectroscopy. Gel images for the Pth1 enzymes used in this manuscript are shown in Figure S1.

For the solubility studies, Pth1 constructs were grown as indicated. Cells were harvested at equal optical density and lysed. Gel samples were taken from the entire cell lysate to show the total protein content. The cell lysate was centrifuged as described in the previous purification references and the supernatant, representing soluble protein, was separated from the pellet which contained the insoluble proteins. Gel samples were prepared from both the soluble and insoluble protein, with the insoluble fraction being resuspended in an equal volume of the buffer as the soluble fraction, so a direct comparison of Pth1 content could be made from band intensity.

### 4.3. Site-Directed Mutagenesis of the S. aureus Pth1 Gate Loop

Mutations were introduced to the pKQV4 plasmid possessing *S. aureus* Pth1 using the QuikChange Lightning (Stratagene, San Diego, CA, USA) site-directed mutagenesis kit following the manufacturer’s protocols and primer design. *S. aureus* Pth1 mutations included E99P, Q100P, and Q102V (*E. coli* Pth1 numbering used throughout). *S. aureus* Pth1 mutants were transformed into chemically competent BL21(DE3)pLys *E. coli* and grown at typical and *S. aureus* Pth1 optimized conditions (both described above).

### 4.4. Pth1 Enzyme Activity

Pth1 activity, or cleavage of substrate peptidyl-tRNA, was evaluated as previously reported [22]. Briefly, the desired amount of Pth1 was incubated for 30 min at room temperature with 1.5 μg of bulk peptidyl-tRNA isolated as described in [49]. The working buffer was 20 mM Tris-acetate pH 8.0, 20 mM magnesium acetate, 40 mM ammonium acetate. The reaction was quenched with a 2X loading dye (100 mM sodium acetate, 35 mM urea, 0.0025 g bromophenol blue, DEPC-treated water to 5 mL total volume) and samples run at 100 V on an 8% acid-urea minigel. After staining with methylene blue, gels were analyzed and substrate cleavage quantitated. In more detail, quantitation entailed calculating the percentage of the cleaved substrate as measured by migration differences observed on acid-urea gels, using the uncleaved control (0% cleaved, 100% inhibited, largest substrate, least migration) and fully cleaved control (100% cleaved, 0% inhibited, smallest substrate, most migration) as bounds [22]. All values were determined as averages of three measurements with the error being the standard deviation. Measurement error, in agreement with results from the previously published method, was less than 5% in all cases. It should be noted that there are two bands in the activity assay. The lower band is the peptidyl-tRNA substrate which can be cleaved and shows a difference in migration distance. The top band in an artifact, presumably the 5S rRNA fragment, that is a remnant of the purification. The 5S fragment does not get cleaved, but is utilized as an internal standard for each lane.

### 4.5. Natural Product Inhibition

Natural product extracts were obtained from tropical rainforest plant material collected in north Queensland, Australia [50,51], Monteverde, Costa Rica [52], Matabeleland, Zimbabwe, and Abaco Island, Bahamas [53]. Plant material was crushed and effused with solvent, see Figure S2. The solubilized fraction was separated from bulk plant material, lyophilized and then stored frozen. Thawed pellets of the crude plant extracts were resuspended in DMSO to form 1% *w*/*v* solutions, the final vehicle for inhibition studies. For inhibition studies, these crude extracts were substituted for water in the previously described Pth1 enzyme activity reactions and all controls were run with volumes of DMSO equal to those in the samples containing natural product inhibitors. Thus the only difference was the presence of natural product extract. It should be noted that Pth1 activity is known to be unaffected by DMSO concentrations exceeding 20% [22], the upper limit used herein. The inhibition reported (Figure 6) was determined as a percentage of inhibited cleavage, described in the previous section. The measurement was an average of three independent measurements. For graphical presentation, the degree of inhibition was binned into the appropriate pentile. As before, all errors were found to be less than 5% as determined from the standard deviation.

### 4.6. Pth1 Kinetic Analysis

Kinetic data were acquired using the same enzyme activity assay described above. The final concentration of Pth1 was 30 μM and all reactions were run at 20 °C. Reactions were carried out at 1:0.13, 1:0.25, 1:0.5, 1:1, 1:2, 1:3, and 1:4 Pth1:peptidyl-tRNA molar ratios. The concentration of the original stock of peptidyl-tRNA was determined using A_260_/A_280_ ratio. The solution was subsequently diluted such that equal volumes of the substrate were added to the enzyme reaction mixture. Reactions were quenched at 5–10 min intervals with ending times (30–50 min) dependent on substrate concentration. Products of the reaction mixtures were separated on acid urea minigels and analyzed as described above. From plots of product vs. time, V_o_ was determined from the initial tangent line slope for each substrate concentration. The assay was repeated in triplicate for all time intervals and peptidyl-tRNA concentrations used. Kinetic parameters were determined using previously reported approaches [54].

### 4.7. Statistical Analysis

Statistical analysis for determining differences in solubility of the expressed *S. aureus* WT and mutants was performed using densitometry. Fraction soluble was calculated from the ratio of the normalized soluble Pth1 band to the Pth1 total band. The results were obtained by analyzing samples from three separate measurements each and used to calculate *p*-values using a two-tailed Student *t*-test. 

Statistical analysis comparing Pth1 kinetic parameters from different clades as well as WT *S. aureus* Pth1 and *S. aureus* E99P Pth1 was carried out using one-way ANOVA analysis with K_cat_/K_m_ and K_m_ values for each enzyme being treated as the independent variables to determine statistical significance between each clade. For both tests, *p*-values smaller than 0.05 indicated a statistically significant difference.

## 5. Conclusions

This report demonstrates the advantage of characterizing multiple homologs, spanning phylogenetic space in antibiotic development. This is particularly relevant for small-molecule inhibition where characterization of only one member of an enzyme family, even one with high homology, may not be adequate. For Pth1, this analysis opens the doors to many different avenues of inhibitor development. Most significant is that both broad and narrow-spectrum inhibition is possible for Pth1. Coupled with phylogenetic characterization and kinetic characterization, the tremendous promise to antibiotic development against this new target is uncovered. The possibility of narrow-spectrum inhibition has the advantages of diminishing the spread of resistance across multiple bacterial species and greater limiting detrimental effects on the host microbiome [55]. Moreover, this and other reports [18,24,25] demonstrate the potential for natural product inhibition targeting Pth1. With a significant number of current therapeutics tracing roots to natural products coupled with the support of the large structural knowledgebase existing for Pth1, novel drug discovery targeting Pth1 is poised for significant breakthroughs. 

## Data Availability

The data presented in this sudy are available on request from the corresponding author.

## Supplementary Materials

The following are available online, Figure S1: Gel Images of Purified Pth1 and Figure S2: Natural Product Extracts.

## Sample Availability

Samples of plasmids are available from the authors.
