# Supplementary material for: Natural Product Inhibition and Enzyme Kinetics Related to Phylogenetic Characterization for Bacterial Peptidyl-tRNA Hydrolase 1
Source: Molecules. 2021 Apr 15;26(8):2281. doi: 10.3390/molecules26082281 (PMC8071115; doi:10.3390/molecules26082281)
Supplement: Supplementary file 1 [file molecules-26-02281-s001.pdf]

Supplementary

Natural Product Inhibition and Enzyme Kinetics Related to Phylogenetic Characterization for Bacterial Peptidyl-tRNA Hydrolase 1

D. Scott Strange [1], Steven S. Gaffin [2], W. Blake Holloway [1], Meredyth D. Kinsella [2], Jacob N. Wisotsky [2], Hana McFeeters [1] and Robert L. McFeeters [1,*]

Fig 1. Gel Images of Purified Pth1 Shown are gels for all purified Pth1 enzymes used in this study. Pth1, indicated by the arrow on the right, was purified using metal chelation chromatography with the resulting fractions labeled.

Escherichia coli Pth1

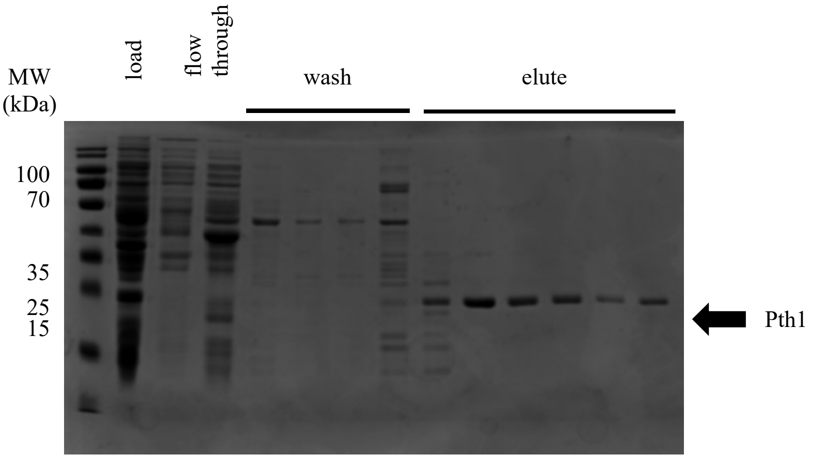

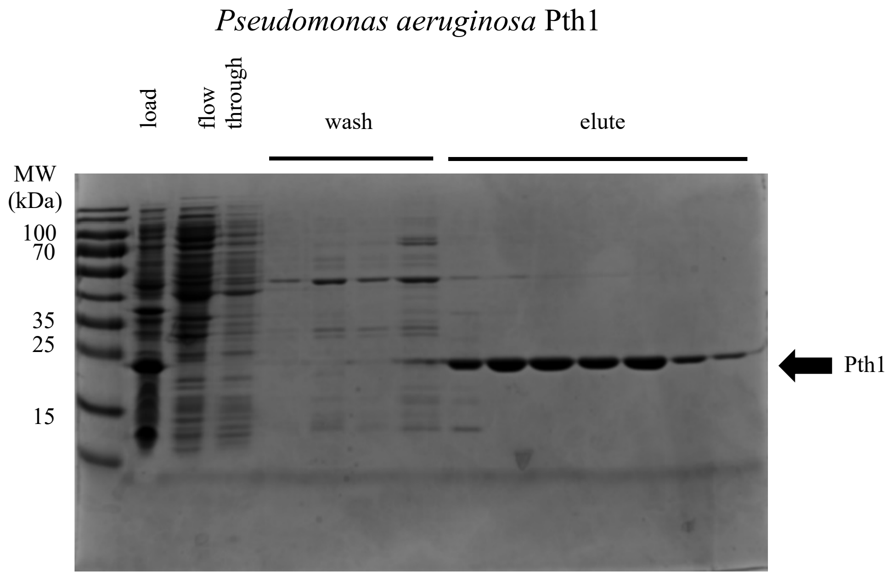

Pseudomonas aeruginosa Pth1
load
flow through
wash
elute
MW (kDa)
100
70
35
25
15
Pth1

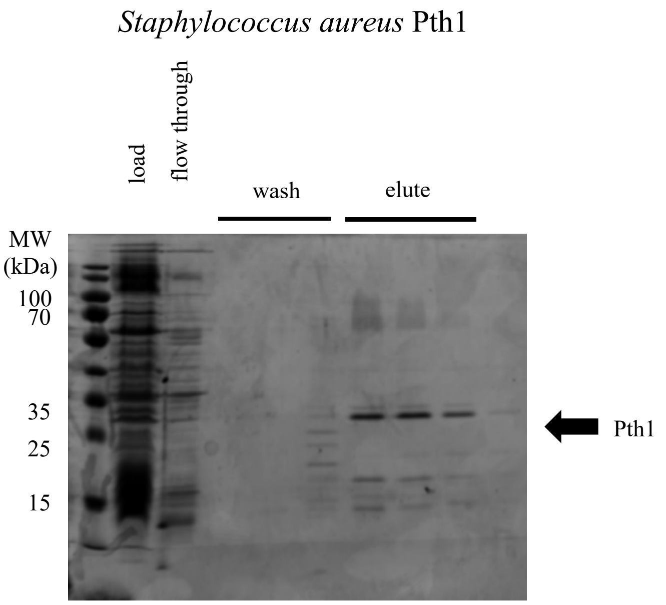

Staphylococcus aureus Pth1
load
flow through
wash
elute
MW
(kDa)
100
70
35
25
15
Pth1

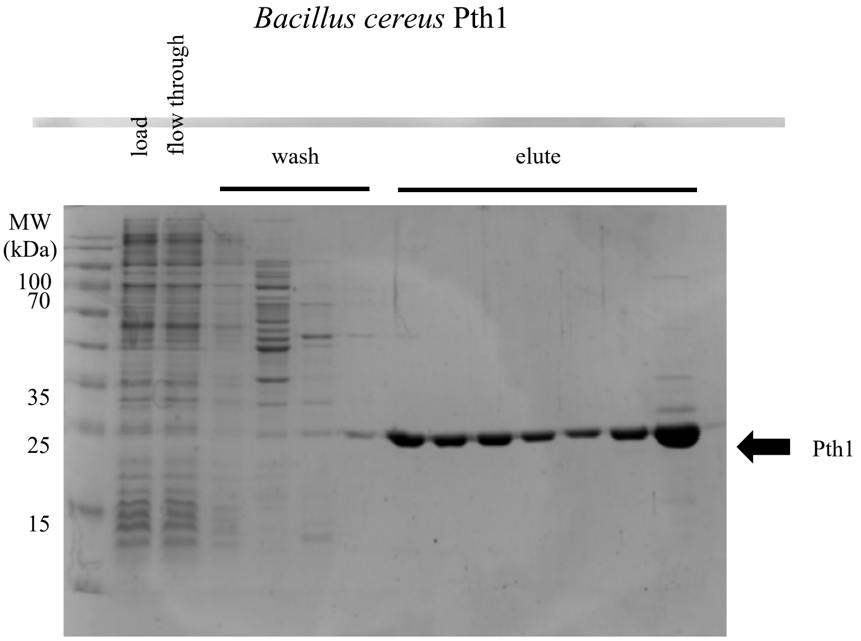
Bacillus cereus Pth1
load
flow through
wash
elute
MW
(kDa)
100
70
35
25
15
Pth1

Salmonella typhimurium Pth1

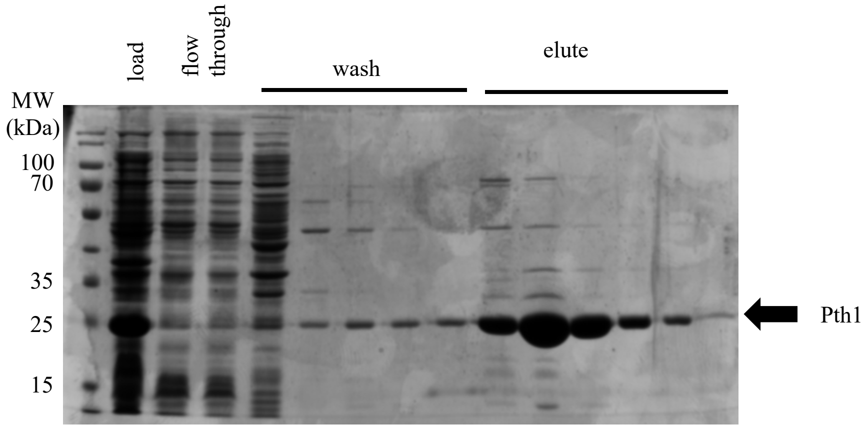

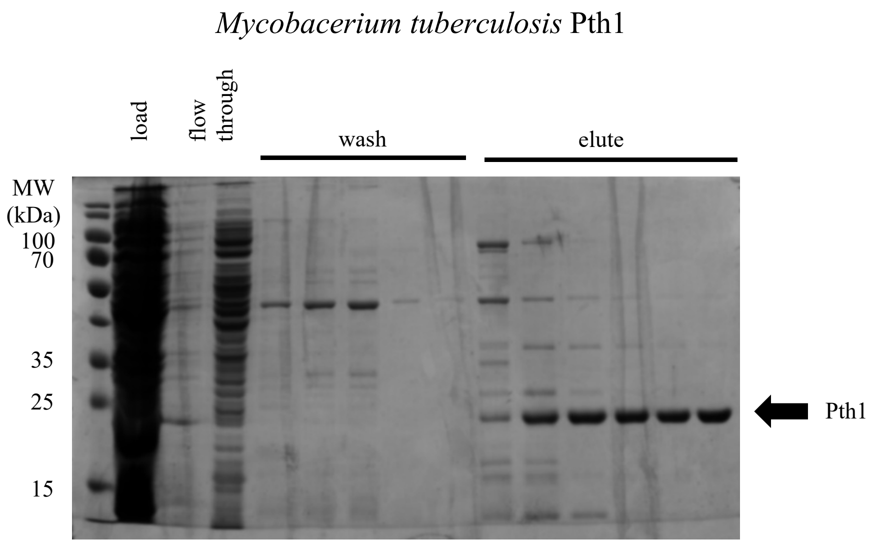

Mycobacterium tuberculosis Pth1
load
flow through
wash
elute
MW (kDa)
100
70
35
25
15
Pth1

Supplementary Fig 2. Natural Product Extracts Shown are the natural products from which the extracts tested for Pth1 inhibition were derived along with the solvent used for extraction.

**1.** ***Ardisia revoluta*** **(bark)**	Acetone
**2.** ***Ardidendron vaillanti*** **(bark)**	EtOH
**3.** ***Mandevilla veraguasenis*** **(bark)**	Acetone
**4.** ***Inga sierra*** **(bark)**	MeOH
**5.** ***Ardisia compressa*** **(bark)**	$CHCl_3$
**6.** ***Exothea paniulata*** **(bark)**	EtOH
**7.** ***Salacia liana*** **(bark)**	$CHCl_3$
**8.** ***Lonchocarpus orotinus*** **(bark)**	EtOH
**9.** ***AlDrymonia conchocalyx*** **(vine)**	EtOH
**10.** ***Cestrum racemosum*** **(bark)**	Acetone
**11.** ***Myorica*** **sp "fuzzy leaf" (leaf)**	Acetone
**12.** ***Drypetes lasiogyna ver australasia*** **(bark)**	$CHCl_3$
**13.** ***Mallotus paniculate*** **(bark)**	EtOH
**14.** ***Albizia adenocephalia*** **(bark)**	DMSO
**15.** ***Ocistea floribunda*** **(bark)**	Acetone
**16.** ***Acacia aulacocarpa*** **(bark)**	DMSO
**17.** ***Conestegba xalapensis*** **(bark)**	DMSO
**18.** ***Urera carcacasma*** **(bark)**	EtOH
**19.** ***Psychotria parviflora*** **(bark)**	Acetone
**20.** ***Sityrax argenteus*** **(bark)**	CH_2Cl_2
**21.** ***Cinnamomum tonduzii*** **(bark)**	EtOH
**22.** ***Syzygium johnsonii*** **(bark)**	EtOH
**23.** ***Ocotea*** **"los llamas" (bark)**	Acetone
**24.** ***Polysoma alangiacea*** **(bark)**	$CHCl_3$/EtOH
**25.** ***Grevilla lilliana*** **(bark)**	$CHCl_3$/EtOH
